# Allergy patient-specific IgE antibody shows significantly stability during 3 months of storage at multiple temperatures from −80 to 25°C

**DOI:** 10.3389/falgy.2023.1239924

**Published:** 2023-09-07

**Authors:** Zhifeng Huang, Huiqing Zhu, Lexin Xiao, Tingting Liu, Hui Gan, Runpei Lin, Wenting Luo, Baoqing Sun

**Affiliations:** Department of Laboratory Medicine, National Center for Respiratory Medicine, National Clinical Research Center for Respiratory Disease, State Key Laboratory of Respiratory Disease, Guangzhou Institute of Respiratory Health, The First Affiliated Hospital of Guangzhou Medical University, Guangzhou, China

**Keywords:** allergy diagnosis, specific IgE (sIgE), stability, allergen, sample storage

## Abstract

The detection of allergen-specific IgE antibodies is an important biomarker for the diagnosis and treatment monitoring of allergic diseases. And the pre-analytical phase is an important part of the overall quality of the laboratory. In this study, 44 patients with allergic diseases (including 23 patients with allergic rhinitis, 12 patients with allergic rhinitis and asthma, and 9 patients with allergic dermatitis) were included in the outpatient center of the Department of Allergy, the First Affiliated Hospital of Guangzhou Medical University. We mixed the serums of the above 44 patients (approximately 0.8 ml of serum volume per patient) into a large volume of serum pool (about 35 ml in total) and divided into 26 parts. And 26 serum samples were stored at 4 different temperatures for 90 days to observe the stability of sIgE antibodies to 16 allergens in serum. The results show that serum sIgE antibody titers in patients with allergic diseases show significant stability during 90 days of storage, even at room temperature. Good stability even after up to 10 freeze-thaw cycles under low temperature storage conditions.

## Introduction

As a collection of biomaterials and accompanying data information, the biological resource database is used by researchers to carry out research on disease mechanisms, clinical diagnosis and treatment, and new drug development, which has greatly promoted the development of medical scientific research. Cryopreservation is one of the indispensable technologies of the biological resource database. In recent years, studies (1, 2) have shown that the concentration and composition of biomarkers may change after long-term storage, which will affect the results of subsequent experiments. Improper storage of biological samples not only causes irreversible effects to the samples, but also may cause higher storage costs. The results of the studies (3–5) showed that most of the biomarkers can be stably preserved below −20°C, while they are significantly affected above 0°C. The effects of cryopreservation on different proteins are different.

Allergen-specific Immunoglobulin E (sIgE) is the major component involved in allergic reactions. The assessment of storage and transportation stability of sIgE in serum is a vital component of pre-analytical quality control in the laboratory. This is also a necessity for creating a serum sample bank for allergic patients. It has been proved that the allergen sIgE in serum can be stored stably at−20°C for 17 days (6), The study by Østergaard et al. found that neither storage at 5°C for at least 10 days nor the type of storage tubes that were frozen for short periods of time affected sIgE levels (7). However, there is a lack of studies on the storage stability of serum allergen sIgE for longer periods of time under different temperature conditions. This study aimed to analyse the effect of storing serum allergen sIgE antibodies under different temperature conditions for intermediate periods. This analysis was conducted to evaluate the preanalytical impact of laboratory tests and to generate additional data for building a biobank for allergic diseases.

## Methods

In this study, 44 patients with allergic diseases (including 23 patients with allergic rhinitis, 12 patients with allergic rhinitis and asthma, and 9 patients with allergic dermatitis) were included in the outpatient center of the Department of Allergy, the First Affiliated Hospital of Guangzhou Medical University. The age distribution was 30 (6.5–48.5) years. Routine detection by the ALLEOS 2000 system (Hycor biomedical, Changsha, China) showed that all the 44 patients were multiple sensitized to common allergens, and at least one allergen-specific IgE was at a high level (sIgE ≥ 3.50 kU_A_/L). Since this study required the same serum to be divided into several portions and stored in different environments for 90 days to monitor changes in sIgE antibodies in the serum, we needed a large volume of serum. We mixed the srea of the above 44 patients (approximately 0.8 ml of serum volume per patient) into a large-volume serum pool (approximately 35 ml in total). Written informed consent was provided from all adult patients or legal guardians of participants under 18 years of age.

**Figure 1:**
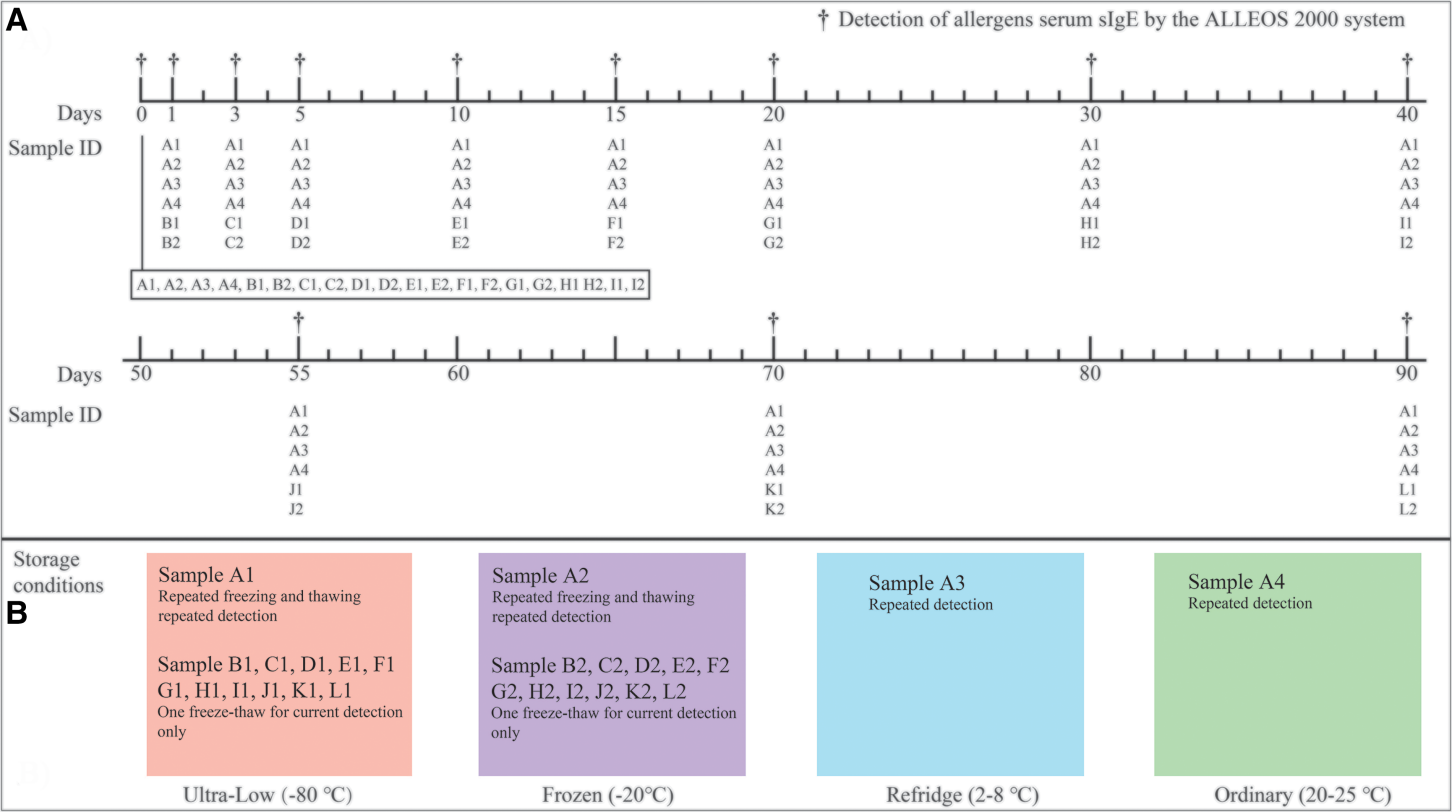
Detection schedule figure (**A**) and different sample storage conditions (**B**). During the 90-day storage monitoring, the fluctuation ranges of the four temperatures were recorded at the same time (the specific temperatures were recorded every morning and afternoon respectively). Records showed that all of the −80°C refrigerators were between −78.5 and −81°C, −20°C refrigerators were between −18 and −21°C, refrigerated refrigerators were between 2 and 8°C, and room temperature was between 20 and 25°C (in an air-conditioned environment) within 90 days.

In this study, a large volume of serological pool samples was fully mixed and divided into 26 parts and named with letters and Arabic numerals respectively. The sample with Arabic numerals 1 was stored at −80°C, including A1-L1 (Ultra-low). Samples with Arabic numerals 2 were stored at −20°C, including A2-L2 (Frozen). In addition, A3 (Refridge) and A4 (Ordinary) are stored at 4–8°C and room temperature (18–23°C) respectively (Figure 1). Since the four samples A1, A2, A3 and A4 will be repeatedly tested (12 times) in 90 days storage, their sample volume is about 5,000 µl. Other samples stored at −80°C and −20°C have a volume of 500 µl because they are only used for one-time testing during storage to avoid the effect of repeated freezing and thawing on IgE antibody concentration.

The ALLEOS 2000 system (Hycor biomedical, American) was selected for the detection of allergen sIgE concentrations in this study due to the advantages of fully automated quantitative detection and very low serum consumption (only 4 µl/test/allergen). The system utilizes magnetic particle chemiluminescence technique. The 16 allergens include *Dermatophagoides pteronyssinus* (Der p, European house dust mite), *Dermatophagoides farinae* (Der f, American house dust mite), *Felis domesticus* (Fel d, Domestic cat), *Canis familiaris* (Can f, Domestic dog), *Equus caballus* (Equ c. Domestic horse), *Gallus domesticus* (G. gallus, Chicken), *Bos domesticus* (Bos d. Domestic cattle), *Charybdis feriatus* (Cha f. Crab), *Penaeus aztecus* (Pan a. Brown shrimp), *Phleum pratense* (Phl p, Timothy), *Alternaria alternata* (Alt a. Alternaria plant rot fungus), *Betula verrucosa* (Bet v. White birch), *Ambrosia artemisiifolia* (Amb a. Short ragweed), *Artemisia vulgaris* (Art v. Mugwort), *Chrysanthemum leucanthemum* (Che l. Marguerite), *Taraxacum vulgare* (Tar v. Dandelion). sIgE levels were expressed in kilo units antibody per liter (kU_A_/L) with the following range: 0.10–100 kU_A_/L. Tests with sIgE levels greater than or equal to 0.35 kU_A_/L were defined as sIgE -positive. SIgE-positive tests were categorized into the following 6 classes: class 1 (≥0.35 to <0.70 kU_A_/L), class 2 (≥0.70 to <3.50 kU_A_/L), class 3 (≥3.50 to <17.50 kU_A_/L), class 4 (≥17.50 to <50 kU_A_/L), class 5 (≥50 to <100 kU _A_/L), and class 6 (≥100 kU_A_/L).

## Results and Discussion

Before cryopreservation of serum samples, 20 samples (A-I) of 26 samples were tested to evaluate the repeatability of the system. The sIgE levels of the 16 allergens included in the study were Der p (31.64 ± 2.2 kU_A_/L), Der f (32.03 ± 1.65 kU_A_/L), Fel d (1.45 ± 0.14 kU_A_/L), Can f (0.44 ± 0.03 kU_A_/L), Equ c (13.68 ± 0.73 kU_A_/L), Gal d (0.99 ± 0.09 kU_A_/L), Bos d (0.37 ± 0.03 kU_A_/L), Cha f (8.31 ± 0.43 kU_A_/L), Pan a (7.43 ± 0.59 kU_A_/L), Phl p (1.64 ± 0.11 kU_A_/L), Alt a (0.21 ± 0.02 kU_A_/L), Bet v (0.4 ± 0.03 kU_A_/L), Amb a (0.2 ± 0.02 kU_A_/L), Art v (0.95 ± 0.06 kU_A_/L), Che l (0.52 ± 0.04 kU_A_/L), Tar v (0.3 ± 0.02 kU_A_/L) (Table 1). They covered different levels between 0.01–40.00 kU_A_/L and comprehensively and systematically evaluated the allergen detection systems used in this study. The results showed that the coefficients of variation of 16 allergens in 20 consecutive tests were between 5% and 10%, indicating that this allergen detection system is very reproducible. According to the industry standard of allergen sIgE detection kit (https://www.nmpa.gov.cn/) issued by the State Food and Drug Administration of China, the fully automatic quantitative detection of serum allergen sIgE instrument with a coefficient of variation of less than 10% is a qualified and excellent product. In addition, all reagents used in this study for the same allergen were produced in the same batch, which would be able to better eliminates reagent errors. Most studies, such as that of Woodhams et al. (8), do not mention the CV of the assay, which may be incorrectly attributed to poor stability due to differences in in detection systems or kits.

**Table 1:** Evaluates the stability of the instrument and operating conditions before the start of storage (0 days).

Allergens	Mean ± SD	CV%
Der p	31.64 ± 2.2	6.96%
Der f	32.03 ± 1.65	5.16%
Fel d	1.45 ± 0.14	9.71%
Equ c	0.44 ± 0.03	5.77%
Can f	13.68 ± 0.73	5.33%
Gal d	0.99 ± 0.09	9.17%
Bos d	0.37 ± 0.03	8.73%
Cha f	8.31 ± 0.43	5.17%
Pan a	7.43 ± 0.59	7.98%
Phl p	1.64 ± 0.11	6.45%
Alt a	0.21 ± 0.02	8.74%
Bet v	0.4 ± 0.03	7.55%
Amb a	0.2 ± 0.02	8.66%
Art v	0.95 ± 0.06	6.71%
Che l	0.52 ± 0.04	7.64%
Tar V	0.3 ± 0.02	7.95%

Der p*- Dermatophagoides pteronyssinus*. Der f- *Dermatophagoides farinae*. Fel d- *Felis domesticus*. Can f- *Canis familiaris*. Equ c- *Equus caballus*. Gal d- *Gallus domesticus.* Bos d- *Bos domesticus*. Cha f- *Charybdis feriatus*. Pen a- *Penaeus aztecus*. Phl p- *Phleum pratense.* Alt a- *Alternaria alternata*. Bet v- *Betula verrucosa*. Amb a- *Ambrosia artemisiifolia*. Art v- *Artemisia vulgaris*. Che l- *Chrysanthemum leucanthemum*. Tar v- *Taraxacum vulgare*.

**Figure 2:**
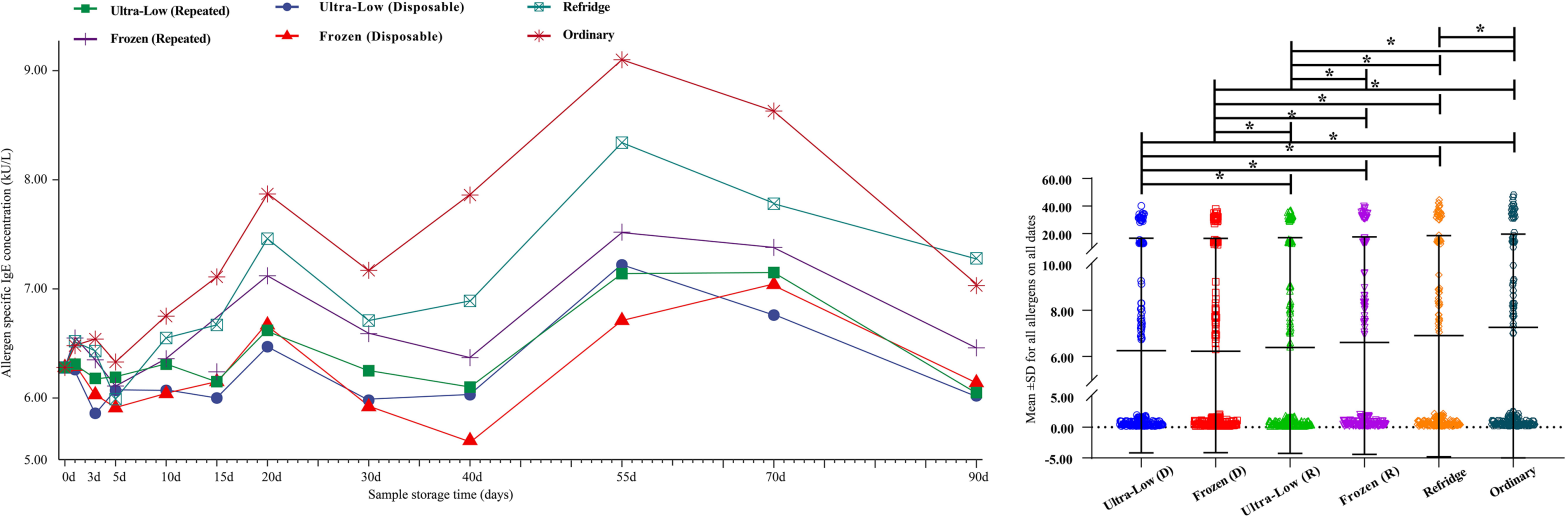
Trends in mean sIgE antibody titers for 16 allergens over 90 days (**A**) and comparison of detection values of all allergens within 90 days under different storage conditions (**B**) d– days. *- *P* < 0.05.

Overall, we integrated the results of 16 allergens and observed the changing trend of the overall level. The average titer of the 16-item allergen sIgE antibody at 0 day was 6.28 kU_A_/L, and with the extension of storage time, the samples stored at −80°C and −20°C showed slight fluctuations (whether repeated freeze-thawed or not), and no significant differences were shown in sIgE antibody titers over a storage time of 90 days (Figure 2A). However, the average titer of sIgE antibody in the samples stored at 4–8°C and room temperature showed an obvious upward trend. This is consistent with the 17 days observation of Rodr í guez-Capote et al. (6), and it is believed that this may be caused by the volatilization of solutes in the serum. In addition, the results of some similar studies support this conjecture (3, 9). However, this deviation did not have a serious impact on the results, as the mean values remained in the same class (Class 3: 3.5 to 17.5 kU_A_/L) even at the maximum offset. Secondly, the differences of the mean values of all allergens detected within 90 days under different storage conditions were compared (Figure 2B). The overall mean values of samples tested in a single run were lower than those of samples repeatedly frozen and thawed under both ultra-low and frozen conditions (−80°C: 6.25 ± 10.42 vs. 6.39 ± 10.66, −20°C: 6.23 ± 10.37 vs. 6.61 ± 11.03) (all *P* < 0.05). Second, the values of the samples stored below 0°C were significantly lower than those stored above 0°C (all *P* < 0.05), regardless of whether they were repeatedly frozen and thawed. The results also showed that the overall mean values of the samples stored under refrigeration were lower than those stored at ambient temperature (6.91 ± 11.71 vs. 7.26 ± 12.39, *P *< 0.05).

**Figure 3:**
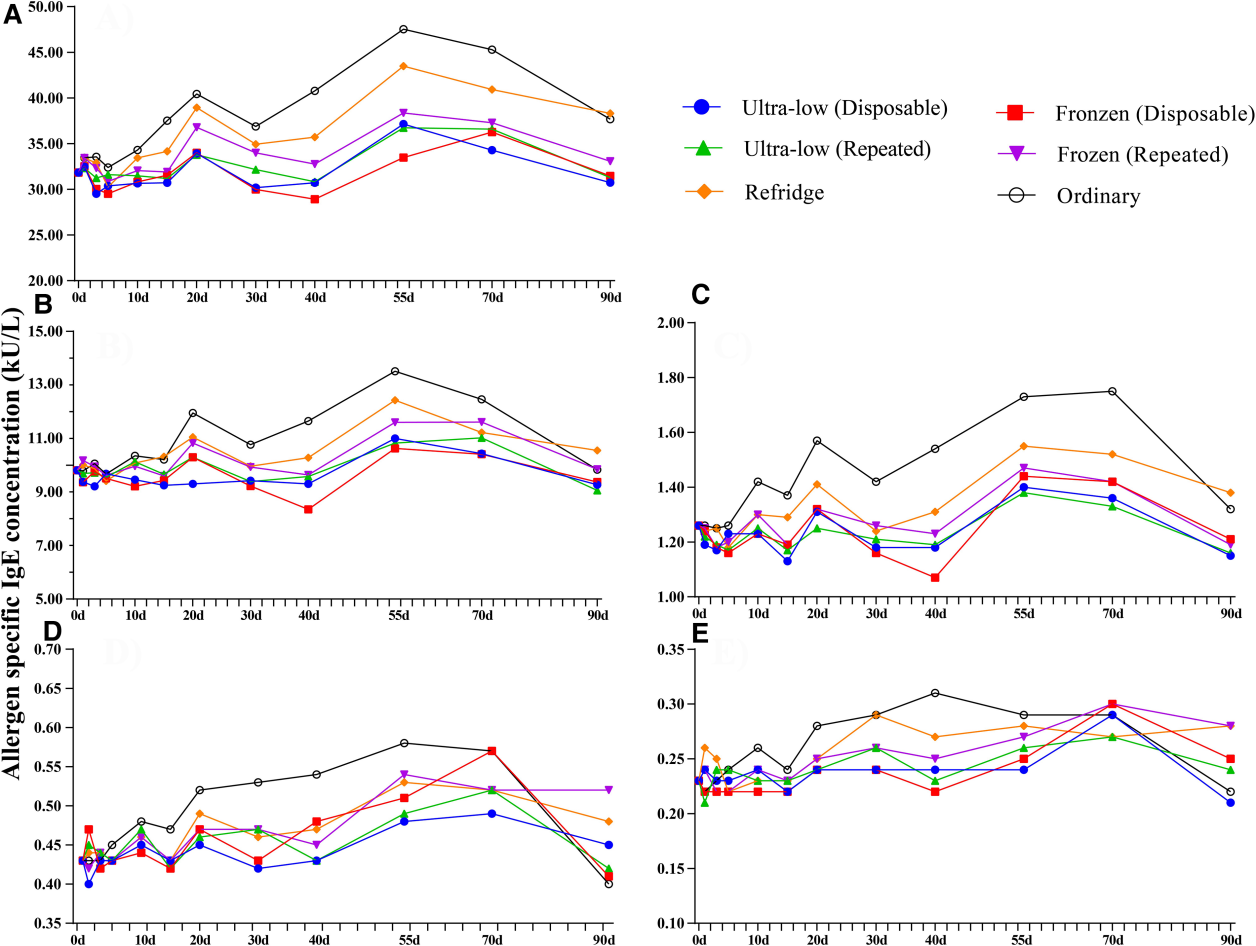
Trends in different allergen levels over 90 days in four preservation environments. (**A**) Class 4: 17.50–50.00 kU/L, (**B**) Class 3: 3.5–17.5.00 kU/L, (**C**) Class 2: 0.7–3.5 kU/L, (**D**) Class 1: 0.35–0.70 kU/L, (**E**) Class 0: 0.10–0.35 kU/L. Ultra-low (**D**)- Ultre-low (Disposable), Ultra-low (**R**)- Ultre-low (Repeated), Frozen (**D**)- Frozen (Disposable), Frozen (**R**)- Frozen (Repeated), d– days.

**Figure 4:**
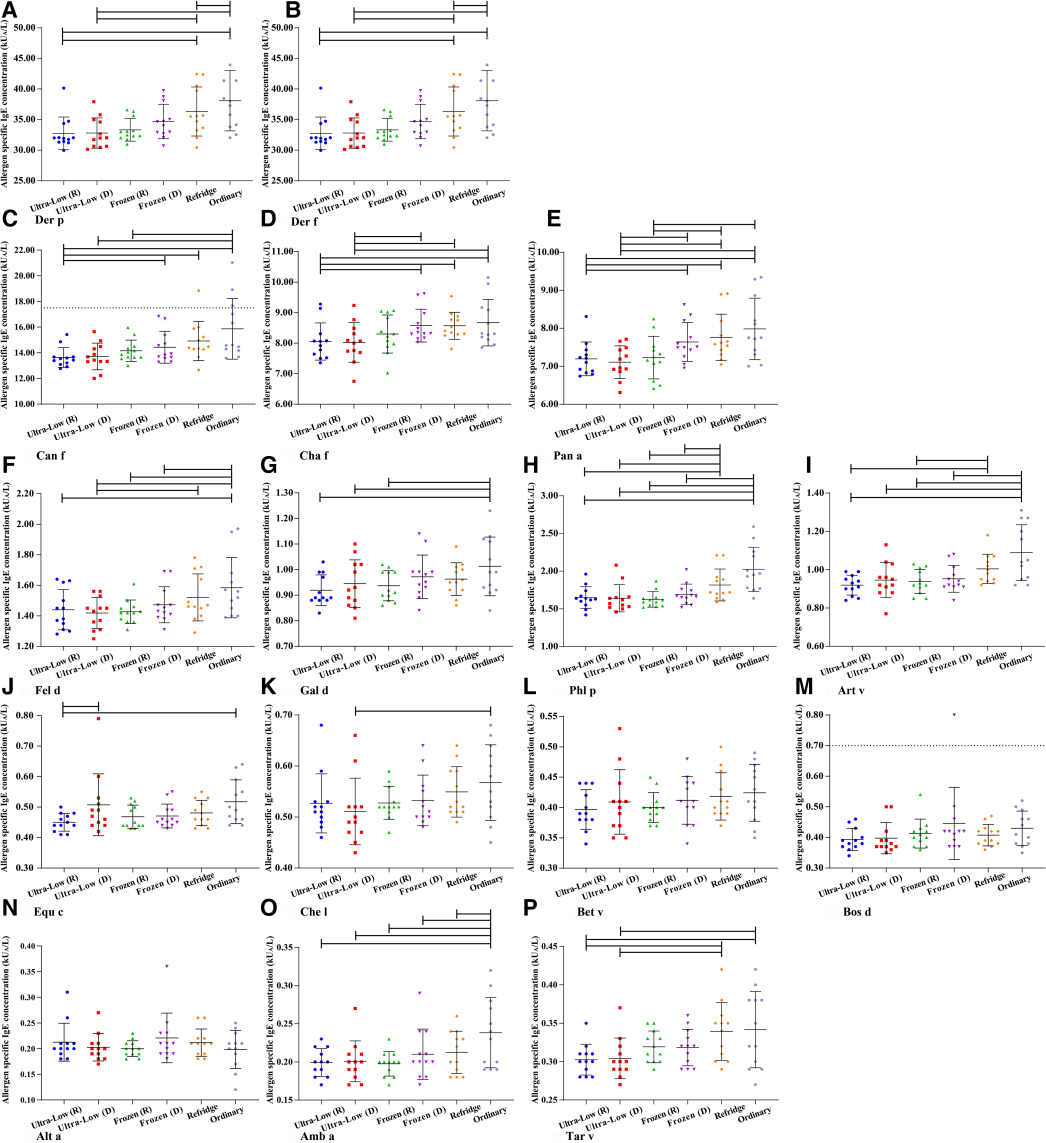
The mean difference of all detection values of a single allergen within 90 days under different storage methods. (**A**) Der p- *Dermatophagoides pteronyssinus*. (**B**) Der f- *Dermatophagoides farinae*. (**C**) Can f- *Canis familiaris*. (**D**) Cha f- *Charybdis feriatus*. (**E**) Pen a- *Penaeus aztecus*. (**F**) Fel d- *Felis domesticus*. (**G**) Gal d- *Gallus domesticus*. (**H**) Phl p- *Phleum pratense*. (**I**) Art v- *Artemisia vulgaris*. (**J**) Equ c- *Equus caballus*. (**K**) Che l- *Chrysanthemum leucanthemum*. (**L**) Bet v- *Betula verrucosa*. (**M**) Bos d- *Bos domesticus*. (**N**) Alt a- *Alternaria alternata*. (**O**) Amb a- *Ambrosia artemisiifolia*. (**P**) Tar v- *Taraxacum vulgare*. d– days.

Based on the definition of serum sIgE classification, we integrated the allergen sIgE results within the same class range and obtained the mean values to explore the stability of different levels of sIgE under different preservation conditions (Figure 3). Similar to the overall average results observed previously, the allergen titers at five levels did not show a particularly significant deviation in 90 days. Slight elevations in samples preserved at room temperature can be observed after 5 days and the titer of allergen in 5–70 days was higher than that in all other storage methods (whether freeze-thaw or not). Room temperature environments appear to be more likely to affect sIgE antibody titers at high levels, while being more stable in the 0.10–0.35 kU_A_/L (Negative) range. The mean difference of all detection values of a single allergen within 90 days under different storage methods were further analyzed (Figure 4). For most of the allergens in this study, the sIgE of the samples stored at −80°C and −20°C within 90 days and without repeated freezing and thawing were significantly lower than those stored at 4°C and room temperature (all *P* < 0.05), and the sIgE of the samples stored at room temperature was the highest (all *P* < 0.05). It is worth noting that for Bet v, Bos d and Alt a allergens, there was no difference in the mean sIgE of allergens stored in various environments for 90 days (all *P* > 0.05).

Cuhadar et al. (1) showed that common clinical chemistry analytes showed adequote stability after up to ten times of freeze-thaw cycle. The result in this study shows that up to 11 times of repeated freeze-thaw in 90 days does not seem to have a large impact on serum sIgE antibodies. For most allergens, there is no difference between samples that have or not have been repeatedly frozen and thawed in ultra-low temperature environments, which is surprising.

## Conclusion

In conclusion, serum sIgE antibody titers in patients with allergic diseases show incredible stability during 90 days of storage, even at room temperature. Cryopreservation is considered to be a more suitable scenario for the preservation of serum from patients with allergic diseases, and it is recommended to use cryopreservation conditions for long-term preservation. Moreover, specific IgE appears stable for up to at least 10 freeze-thaw cycles.

## Data Availability

The raw data supporting the conclusions of this article will be made available by the authors, without undue reservation.
